# Comparison between current and ideal condition of educational justice from the students’ viewpoints at Jahrom Nursing and Paramedical School

**Published:** 2014-01

**Authors:** MOHAMMAD ALI MONTASERI, MOHSEN HOJAT, MAHDI KARIMYAR JAHROMI

**Affiliations:** Jahrom University of Medical Sciences, Jahrom, Iran

**Keywords:** Education, Justice, Ideal, Current

## Abstract

**Introduction**: Educational justice is a process by which all those involved in education are pondering and seeking to establish it in their regulatory environments. This study aimed to investigate effective factors in an ideal educational justice and the current condition of educational justice from the students’ viewpoint and ultimately increase the awareness and understanding of authorities and educational planners of the existing shortcomings.

**Methods**: This is a descriptive-analytical study. Samples include all nursing, operating room, and anesthesia students of nursing and paramedical college who had passed at least 5 semesters. Data collection was carried out through a scholar questionnaire. Validity was assessed through content validity and reliability of the questionnaire was evaluated using a pilot study.  In order to determine the status of the scores, 5 points (very high), 4 (often), 3 (moderate), 2 (low) and 1 (very low) were assigned, respectively. To determine the justice level, a 35 score interval was considered as very low, low, medium, high and very high. SPSS software, descriptive statistics, independent t-test and ANOVA were used to analyze the data.

**Results**: There was a significant difference between the ideal and the current conditions in all items (p≤0.001) and also in the total mean score of ideal condition  and mean score of current condition (p=0.010).

**Conclusion**: In an educational system, educational methods and aims should be regulated in a way that principles and components of justice are attainable and distribution and allocation of educational facilities of justice are considered thoroughly.

## Introduction

Justice means equity and removing discriminations over inequality in social facilities, but differences which root in qualification, talent, and activities of individuals should be maintained (1). Educational justice is a process by which all those who involve in education are pondering and seeking to establish it in their regulatory environments. But since it has not been considered thoroughly, public belief is that public access makes educational justice. However, educational justice has a broader meaning and includes all conditions, potential and actual facilities, educational setting and process considering individual talents and capabilities (2, 3). Several definitions for the educational justice have been stated and one of which is providing a good opportunity in the educational systems. Naturally, if the students were identical, equal opportunities and equal distribution of educational resources would mean justice. But the differences between students in different aspects create differences in educational needs and a set of questions in this area (4). Thus, educational justice should be considered based on vertical and horizontal justice. Horizontal justice in training of medical sciences is equal to the students and scholars who possess the same IQ and educational capabilities but studying in different universities and are able to access the same research and educational facilities. Vertical justice in training of medical sciences is equal to various and different methods of teaching so that scholars and students are able to have thorough and correct understanding of the contents. In addition, we would have different expectations with different educational capabilities and facilities from individuals (5). Therefore, to understand our possessions, wishes, and the gap between them which compose our needs, conventional methods of assessment and evaluation are usually applied (2). This is also taken into consideration as an important factor in distinction of students. Several studies have been conducted to determine how to enforce justice in education (6). The results of some studies reveal that inequality in access and distribution of educational facilities is severe in developing countries (7). The study of Mohebbi et al. aimed to investigate and provide a tool for assessing justice in educational environments of health systems in universities of medical sciences. Factors such as the level of students’ satisfaction, faculty, and staff, faculty recruiting conditions, availability of educational space, required academic resources, campus facilities, internet access and databases, students’ per capita, the proportion of research, cultural and administrative role of faculty to teaching role, presence of educational curriculum revision, the proportion of educational environment to the faculty and students, and plans for promoting the quality of educational services and so on were emphasized in investigating educational justice (8).

The study conducted by Momayezi et al. entitled “investigating justice in clinical training from the viewpoints of nursing and midwifery students” demonstrated that only 3.3% of the students evaluated the current condition of clinical educational justice as very satisfactory and about %50 estimated justice in distribution of facilities of clinical centers and using highly educated and experienced clinical instructors as satisfactory, while the mean of justice which is the most important principle of education was at a medium level. Finally, researchers considered policymaking and respecting students’ opinion in planning to achieve educational justice highly essential (9). This study was carried out to investigate the effective factors in an ideal educational justice and the current condition of educational justice from the students’ viewpoints and finally to increase awareness and understanding of authorities and educational planners of the present shortcomings.

## Methods

This descriptive-analytical study was conducted to investigate and compare the current and ideal condition of justice in education from the viewpoints of nursing and premedical students at Jahrom University of Medical Sciences. The samples were selected from all nursing, operating room (OR), and anesthesia students of nursing and paramedical school at Jahrom University of Medical Sciences who had passed at least 5 semesters (113 students). To collect data, a scholar questionnaire was applied consisting of 5 areas such as planning, implementation, evaluation, resources and students along with 35 closed-ended questions on Likert scale. Students were assured that the checklists would remained anonymous and their contents confidential.

The validity and reliability of the questionnaire were evaluated through content validity and a pilot study respectively in which 20 operating room students participated and Cronbach's alpha coefficient reached 91% for the whole questionnaire and over %70 for each area. The data was collected by the students. In order to determine score status, score 5 (very high), 4 (high), 3 (moderate), 2 (low), and 1 (very low) were assigned. To determine the mean score, the score of each item was multiplied by its frequency and the result was divided by the total number of persons (113). To determine justice level, a 35-point interval was considered as very low (1-35), low (36-70), moderate (71-105), high (106-140), and very high (141-175).

Also, regarding scores of each item, the score below 2 was considered as “below moderate”, 2-3 as “moderate”, 3-4 as “desired”, and over 4 as “great”. SPSS 14 (SPSS Inc, Chicago, IL, USA), descriptive statistics (mean, standard deviation, percentages), independent t-test and ANOVA were used to analyze the data (p<0.05).

## Results

The mean age of the participants was 21.29±2.53 years, with 74(%65.5) being female and 39(%34.5) male. Considering the field of study, 72(%63.7) were nursing students, 20(%17.7) operating room students, and 21(%18.6) anesthesia students. Total mean±SD of grade point average (GPA) of the participants was 15.79±0.93.

The results obtained from this study indicated that there was a difference between the acquired mean±SD scores of the ideal condition reported by women [135.16±30.12 (good)] and men [91.77±33.70 (moderate)] (p=0.001).

Considering field of study, the acquired mean of current condition of nursing students 73.14±21.36, OR students 91.20±19.13, and anesthesia students 85.62±20.38 were reported which indicate a significant difference (p=0.001). Also, there was a significant difference between the mean score of current condition of nursing and OR students (p=0.001) and nursing and anesthesia students (p=0.017). But there was no significant difference between OR and nursing students (p=0.393). The mean of acquired score of ideal condition of nursing students 109.60±37.95and the OR students 143.55±21.06 and anesthesia students 134.24±34.41 was reported which indicates a significant difference (p=0.001). There was a significant difference between the mean of score ideal condition of nursing and OR students (p=0.001) and nursing and anesthesia students (p=0.005). But the difference between OR and anesthesia students was not significant (p =0.396).

The following tables shows the mean of ideal and current condition based on sex and field of study.

**Table 1:** Comparison of mean score of ideal and current condition based on sex

**Condition**	**Sex**	**Mean±SD**	**Independent t-test**	
			**t**	**p**
**Ideal**	**Female**	135.16±30.12	-3.75	0.001
	**Male**	91.77±31.70		
	**Total**	120.19±37.5		
**Current**	**Female**	80.11±22.40	0.41	0.335
	**Male**	75.90±21.11		
	**Total**	78.65±21.96		

**Table 2:** Comparison of mean score of ideal and current condition based on field of study

**Condition**	**Field of study**	**Mean±SD**	**ANOVA test**	
			**F**	**P**
**Ideal**	**Nursing**	109.60±37.95	4.773	≤0.001
	**Operation room**	143.55±21.60		
	**Anesthesia**	134.24±34.41		
	**Total**	120.19±37.50		
**Current**	**Nursing**	73.14±21.36	3.064	≤0.001
	**Operation room**	91.20±19.13		
	**Anesthesia**	85.62±20.38		
	**Total**	78.65±21.96		

Acquired by the students with the GPA A(17-20) equal to 135.93±35.81, and the students with the GPA B(15-16.99) equal to 120.24±38.33 and the students with GPA C(12-14.99) equal to 101.42±24.25 was reported which does not indicate any significant difference (p=0.064).

But there was a significant difference between mean of ideal condition in the groups A and C(p=0.019). 

The acquired mean of current condition by the students with the GPA A(93.86±24.14), students with the GPA B(75.93±21.42), and the students with the average GPA C(80.67±16.62) was reported which reveals a significant difference (p=0.016). Comparing the mean between two groups of A and B, there is a significant difference (p=0.004), but it was not significant between other groups. The following table shows the mean of ideal and current justice status in various areas from the students’ viewpoints.

**Table 3:** Comparison of mean of ideal and current conditions of educational justice in various areas

**Area**	**Ideal condition** **Mean±SD** **(N=113)**	**Current condition** **Mean±SD** **(N=113)**	**t-test**		
			**t**		**p**
**Education (10 items)**	3.36±1.21	2.17±1.19	2.04	0.001	
**Evaluation (4 items)**	3.19±1.51	1.84±1.25	0.95	0.001	
**Managing and administration (10 items)**	3.52±1.43	2.38±1.27	1.57	0.001	
**Educational references (5 items)**	3.54±1.46	2.43±1.37	2.64	0.001	
**Student and welfare (6 items)**	3.44±1.53	2.2±1.29	1.24	0.001	
					

As Table 3 indicates, the students’ viewpoints, proportion educational plans with the students’ sex, condition of selecting instructors and courses, selecting supervisors, performing evaluation process, applying tests which actually measure clinical and practical skills of the students, evaluating instructors based on performance quality, flexibility of instructors against constructive criticisms and accepting suggestions, providing equal material and spiritual (horizontal justice) incentives (facilities), providing especial incentives for top students (vertical justice) below moderate level (mean score below 2), determining educational goals for both students and instructors (providing lesson plan) with the mean score of 3.10±1.41 in high level and other cases were assigned in moderate level.

From the students’ viewpoints, the proportion of educational plans to students’ sex reported the mean score of 1.28±1.41 (the least impact) and performing evaluation process (performing evaluation of educational system by the people out of the system) which were in average level, and appropriate library facilities and access to reference books with the average score of 4.04±1.22 were in high level (the highest impact). Other options in high levels also cause establishing justice in education (ideal condition). 

Totally, the mean score of ideal condition was 120.19±37.5 (high level) and the mean score of current condition was 78.65±21.96 (moderate level) which reveals a significant difference comparing their means (p=0.001).

## Discussion

Educational justice is one of the most challenging discussions in education. Creating a stress-free learning environment, providing students’ growth, implementing the same rules and regulations for all, providing background to achieve skills and preparation for the labor market are of educational justice issues (10).

“Proper library facilities and access to reference books”, “proper benefiting from educational aids and technology”, “active presence of instructor during field training” in this study were the most remarkable desires of the students to achieve ideal conditions. The major concern of the current condition refers to the items “the condition for choosing the supervisor”, “the condition for choosing instructors and the courses”. Considering the total areas, the most impact is allocated to “managing and administration” and the major concern refers to “evaluation area”. In line with this, the results of the study of Mazloomi et al. (2001) indicated that the best possible strategy to establish educational justice in the ideal condition was to match the teaching methods with the students’ competence, have access to various books and educational journals, and increase the quality of educational pamphlets (10).

The most important strategies to establish justice in educational system were also observing class time, giving students the authority to choose the instructor and supervisor, a careful evaluation of mid-term and final exams, observing meritocracy in choosing top students, clarifying how to compose and when to evaluate, and fair distribution of educational facilities which are close to the results of the present study (11).

One of the resources which is in the authority of educational system as input and an instrument to measure injustice is experience, expertise and academic qualifications of instructors and educational staff (12). The results of this study indicated that scientific competence and the skills of instructors and the application of proper teaching methods by the instructor were the most important demands of the students to achieve ideal condition of educational justice. In the study conducted by Mazloomi (2001) investigating the qualities of a good instructor, the most important qualities were expressed as respecting students, mastery over the teaching issue, having lesson plan, having religious beliefs, and observing justice (10). It can be concluded that students are always seeking justice from the teacher side and express it as the remarkable quality of a good instructor.

In the study of Sanagou et al. (2001), students expected the instructors to respect them equally regardless of student shortage (13). The results of the study by Zadegan et al. (2001) indicated that from the students’ viewpoints, observing justice and commitment regarding the students was a more remarkable quality than others. Thus, the introduction of education depends more on the characteristics and qualities of the instructor which affect all aspects of education (14).

Evaluation is a dynamic process through which skills and education quality evolve. When the students fail achievement and feel that learning occurs just for some of the students, and this teaching and evaluating method would not guarantee professional qualification, achievement of professional life, or continuation of study, they feel inequity (15). The results of this study showed that “evaluation area” had the lowest score in current condition of educational justice. In the study of Golparvar and Nadi (2009) justice in evaluating students was associated with the negative stress of middle school students (16). In the study of Sanagou et al. (2011), the students expressed that considering the fact that all the students are equal in bedside performance, or even lower, the evaluation by the instructors was unfair. This is where the students are subject to injustice in educational environment which could be solved by performing procedural justice (13).

Javadian et al. (2003) emphasize the appropriateness of the scientific contents with the learners’ ability (17). Also, the results of the study of Mesrizadegan (2011) indicated that the present tests could evaluate neither the students fairly nor the changes in educational behavior, nor differentiate weak students from the strong ones (18). As Cohen-Charash and Spector (19) and Colquitt (20) argue, understanding the principles of justice brings about increasing commitment of the people to their organizational goals. Consequently, the sense of worth logically, ensuring of positive results commensurate with the effort and competence should be linked with academic commitment as well (21).

The results of the present study demonstrated that in the area of managing and administration, “flexibility of the instructors against constructive criticisms and accepting the suggestions” and “scientific and technical competence of the instructor” from the viewpoints of the students was expressed as important factors in achieving ideal justice and was the major concern of the current condition.

In the study conducted by Aghakhani et al. (2011), correct teaching of theory, correct clinical teaching, and accepting criticism were the highest instructor priorities in professional ethics from the viewpoints of the students (22). In analysis of the economy of education, the main criteria for allocating resources and benefiting from the training were efficiency, equity and justice. Justice in educational resources was considered the most effective factor in creating the ideal condition from the students’ viewpoints in this study (23).

In the study carried out by Adhami et al. (2002), in investigating the relationship between educational facilities and human resources in departments of basic sciences with the academic achievement of medical students, there was a positive correlation between educational facilities and human resources in the departments of basic sciences and also instructors’ academic ranks and students’ academic achievement with the scores of inter-sectional and basic sciences (24). Askari (2005) investigated the sources of motivation loss among the university students from their viewpoints and found that the students’ dissatisfaction with unequal educational facilities in all departments caused noticeable motivation damage. According to Azimi et al. (2011), development of IT and communication promote justice in medical education (25). In contrast to these findings, Mesrizadegan et al. (2010) in investigating effective factors in achieving educational justice from the viewpoints of the dentistry students found that making infrastructures and creating equal facilities for the academic and educational achievement were less important in comparison to others (18). In many cases, feeling of violating rights causes uncertainty and anxiety among the people. If people in these cases undergo justice, they will easily involve themselves in behavior beyond formal students’ roles by getting rid of uncertainty and anxiety (26).

## Conclusion

Injustice in the area of behavior and group interaction undermines the feeling of commitment and attachment to organizational and personal goals since individuals’ belonging and commitment even to their personal goals is an exchange process. Thus, the individual makes an attempt for their personal goals. Then they apply the feedbacks they receive from the conditions and the environment as reinforcing or weakening factors in motivation related to their goals. Therefore, lack of justice disturbs the equation related to achievement and appropriateness and by doing so the level of students’ academic commitment is undermined (27). Thus, it is better to regulate educational methods, goals, and axis in an educational system so that principles and components of justice are accessible. In these cases attention should be paid to distribution and allocation of educational facilities in order to fulfill comprehensive justice. Accordingly, achieving educational justice requires multilateral efforts and long term plans. Overcoming cultural, social, and economical inadequacy, fair distribution of budgets and educational facilities, granting scholarship and academic costs based on removing inequity with defined principles, and also commitment to measure and analysis of proposed indicators in universities of medical sciences following control and decision making regarding removing inequities and academic conditions are the measures which can pave the way to achieve educational justice in educational system in higher education (1).

A limitation of this study was the lack of a standard list of codes and a checklist that was relieved with using multiple sources of credible and competent survey of teachers. Also Students were unfamiliar with some concepts such as "vertical and horizontal equity" which was resolved by providing the necessary explanations and answers to questions. 
